# Emergence of Echinocandin Resistance Due to a Point Mutation in the *fks1* Gene of Aspergillus fumigatus in a Patient with Chronic Pulmonary Aspergillosis

**DOI:** 10.1128/AAC.01277-17

**Published:** 2017-11-22

**Authors:** Cristina Jiménez-Ortigosa, Caroline Moore, David W. Denning, David S. Perlin

**Affiliations:** a Public Health Research Institute-New Jersey Medical School-Rutgers Biomedical Health Sciences, Newark, New Jersey, USA; b Mycology Reference Centre, Manchester, University Hospital of South Manchester, Manchester, United Kingdom; c University of Manchester, Manchester, United Kingdom

**Keywords:** Aspergillus fumigatus, chronic pulmonary aspergillosis, echinocandin resistance, *fks1* mutation, micafungin

## Abstract

We have identified the first case of an *fks1* hot spot 1 point mutation causing echinocandin resistance in a clinical Aspergillus fumigatus isolate recovered from a chronic pulmonary aspergillosis patient with an aspergilloma who first failed azole and polyene therapy and subsequently failed micafungin treatment.

## TEXT

Invasive aspergillosis (IA) and chronic pulmonary aspergillosis (CPA) are both life-threatening mycoses mainly caused by the fungal pathogen Aspergillus fumigatus. Triazole antifungal drugs represent first-line therapy for all forms of aspergillosis. Voriconazole is used as the primary azole for treatment of IA, while both itraconazole and voriconazole are used to treat CPA; posaconazole is more often used for prophylaxis (1, 2). Even though acquired resistance during therapy is rare, epidemiological studies have shown that Aspergillus azole resistance has increased over the past decade in patients with prolonged exposure to azoles, notably those with CPA (3). Mutations in the *cyp51A* gene leading to amino acid substitutions in the target enzyme lanosterol 14α-demethylase are the major mechanism of azole resistance (1, 4, 5), yet non-*cyp51A*-mediated resistance has been recently reported (6).

Micafungin (MCF) is a member of the echinocandin class of antifungal agents that targets the fungal cell wall by the inhibition of the β-(1,3)-glucan synthase (GS), an enzyme unique to fungi and responsible for the synthesis of the major cell wall component, β-(1,3)-d-glucan. MCF has demonstrated *in vitro* and *in vivo* activity against A. fumigatus, as well as synergistic activity when used in combination therapy with azoles (isavuconazole, itraconazole, or voriconazole) or with amphotericin B (7, 8). The first-in-class echinocandin, caspofungin, was initially approved by the FDA to treat patients with IA refractory to conventional therapy. Subsequently, micafungin has also been used to treat patients with IA as a second-line drug. Echinocandin resistance in Candida spp. has been linked to mutations in the *FKS* genes, which encode the β-(1,3)-glucan synthase enzymes (9–11). In A. fumigatus, an engineered point mutation in the hot spot 1 region of the *fks1* gene was also sufficient to confer resistance to echinocandin drugs (12). Yet, to date, no echinocandin-resistant A. fumigatus isolates harboring a characteristic *fks* mutation have been recovered from patients after exposure to an echinocandin. In this study, we report the first case of an *fks1* hot spot 1 point mutation causing echinocandin resistance in a clinical A. fumigatus isolate recovered from a chronic pulmonary aspergillosis patient who initially failed azole and polyene therapy and subsequently failed echinocandin therapy.

**FIG 1:**
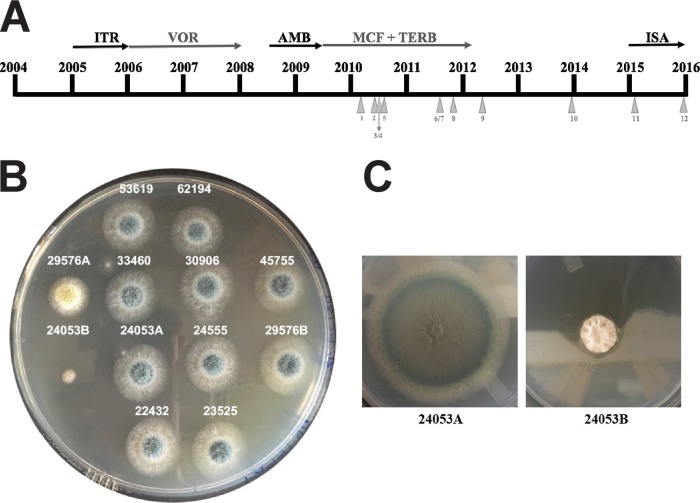
(A) Timeline for patient antifungal therapy. Collection of the Aspergillus species isolates for the current microbiological study is shown (triangles). (B) Isolates recovered from the patient grown for 2 days at 37°C on a PDA plate. (C) A. fumigatus clinical isolates 24053A and 24053B grown for 4 days at 37°C on PDA plates. Isolate 24053B grows at a lower rate and sporulates very poorly compared to the rest of isolates collected from the patient.

A 66-year-old lifelong-nonsmoking female complained of weight loss, fatigue, and severe breathlessness. The patient had severe kyphoscoliosis as a child, which was treated with the insertion of spinal rods in early adulthood. She had suffered recurrent chest infections for many years. She first presented in 2001 with an irritating cough, and several treatments with antibiotics failed to alleviate it. After 2 years, the cough worsened. The patient then coughed up large amounts of blood (hemoptysis), requiring intensive care admission; this was treated with embolization and oral tranexamic acid. She continued to cough and produced green sputum and lost weight. Her Aspergillus precipitin (IgG) titer was high, and a computed tomography (CT) scan demonstrated chronic cavitary pulmonary aspergillosis with a large fungal ball (aspergilloma). She started itraconazole therapy in 2005 (200 mg twice daily [BID]) but failed to respond despite satisfactory blood drug levels, and she was switched to voriconazole in 2006 (150 mg BID). Considerable improvement was seen initially, and the patient gained weight. Voriconazole therapy continued for 2 years. However, the Aspergillus precipitin titer remained high, and the cough continued. Further tests showed that trough plasma levels of voriconazole were above 0.5 mg/liter; however, the Aspergillus fumigatus isolates recovered were resistant to itraconazole, voriconazole, and posaconazole. She received intravenous (i.v.) amphotericin B (120 mg daily) for 3 weeks without any impairment of renal function. She had further intermittent courses of therapy with amphotericin B at the same daily dose without improvement over the following year. In June 2009, the patient started i.v. micafungin at 150 mg 6 times weekly with oral terbinafine (250 mg BID). She improved substantially and continued to take micafungin along with terbinafine to minimize the risk of resistance. She remained on this combination until she developed more hemoptysis, and therapy was discontinued in January 2012. She remained off therapy for over 2 years. Hemoptysis recurred, and she was trialed on isavuconazole in 2015/2016 (200 mg three times daily for 2 days and then 200 mg a day). A timeline of the antifungal therapy of the patient is shown in Fig. 1A.

Once micafungin therapy was initiated, 12 consecutive Aspergillus species isolates (11 A. fumigatus and 1 A. flavus) were recovered from sputum cultures (Fig. 1B) and were submitted for antifungal susceptibility testing in accordance with the guidelines described in CLSI document M38-A2 (13). The drugs used were isavuconazole (ISA; Astellas Pharma USA, Inc., Northbrook, IL), itraconazole (ITR; Sigma-Aldrich, St. Louis, MO), voriconazole (VRC; Pfizer, Inc., New York, NY), posaconazole (POS; Merck Sharp & Dohme Corp., Rahway, NJ), amphotericin B (AMB; Sigma-Aldrich), caspofungin (CSF; Merck Sharp & Dohme Corp.), micafungin (MCF; Astellas Pharma USA, Inc.), and terbinafine (TERB) (Novartis Pharmaceuticals, East Hanover, NJ). Multilocus sequencing typing (MLST) of the 11 A. fumigatus strains collected from the patient was conducted (14). The promoter and open reading frame of the *cyp51A* (Afu6g12400) and the *fks1* (Afu4g06890) genes were sequenced to identify mutations that account for resistance (Table 1).

**TABLE 1:** Oligonucleotides used for *fks1* and *cyp51A* PCR and sequencing in A. fumigatus

No.	Oligonucleotide name	Sequence 5′→3′	Purpose
1	AfFKS1 −751F	CCTGAGTTGGTGGTCAAT	Af*fks1* PCR amplification
2	AfFKS1 6378R	GACTGGCGAAACACGTTG	
3	AfFKS1 −211F	CTGCGACTCGAGATTCAG	
4	AfFKS1 697F	GCATGCGCAACATGTATG	
5	AfFKS1 1562F	CGCACAATCGCTTTACAC	
6	AfFKS1 1947F	CGTCAGTATGTGGCTAGC	Af*fks1* sequencing
7	AfFKS1 2405F	GATTTCTCAAGTTTGGAATGC	
8	AfFKS1 3043F	CGATCAAGCTCCTGTACC	
9	AfFKS1 3401F	GTCTGACAACCAGAATCAC	
10	AfFKS1 4269F	GTCCAGGAACTGACAGAG	
11	AfFKS1 5117F	CGAAGTCATGTTCTTCCTTG	
12	AfFKS1 5931R	CTTCGAGGCGCTGGATAC	
13	AfCyp51A −991F	CGTCGATCTGTGTGACAC	Af*cyp51A* PCR amplification
14	AfCyp51A 1993R	CTAGAAGGAGCAGGACTG	
15	AfCyp51A −819F	CATGCTGGGAGGAATCTC	
16	AfCyp51A −147F	GCTGGTCTCTCATTCGTC	Af*cyp51A* sequencing
17	AfCyp51A 502F	AGAGTCTCATGTGCCACT	
18	AfCyp51A 1151F	CACTCCTCTATTCACTCTATC	

**FIG 2:**
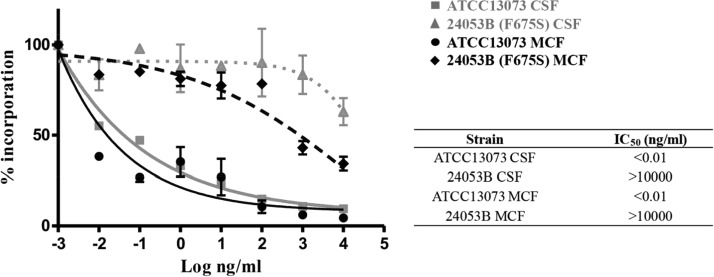
Echinocandin kinetic inhibition profiles for wild-type ATCC 13073 and clinical isolate 24053B. Product-entrapped β-(1,3)-glucan synthase complexes were assessed by incorporation of [^3^H]-UDP-glucose into radiolabeled product and evaluated using a sigmoidal-response (variable-slope) curve. Echinocandin inhibition kinetics yielding 50% inhibitory concentrations (IC_50_s) are expressed in nanograms per milliliter.

Twelve isolates were collected from the patient after MCF treatment; 11 of them were classified as A. fumigatus, and 1 isolate was classified as A. flavus after internal transcribed spacer PCR (ITS-PCR) identification. All A. fumigatus isolates but one (24053B) grew well on potato dextrose agar (PDA) plates, showing the typical dark-green colonies (Fig. 1B); in contrast, 24053B presented a diminished growth rate and a reduction in sporulation, hence, its white colony color (Fig. 1C). To assess the genetic diversity and the potential relatedness of the A. fumigatus isolates recovered from the patient, MLST was carried out, since a previous study in patients with aspergilloma reported the extreme genetic diversity in isolates recovered from fungal balls (15). MLST revealed only two different sequence types (STs), ST9 and ST12 (Table 2). According to the CLSI epidemiological cutoff values (ECVs) (16), all isolates except one (45755) were classified as wild type (WT) for all the triazole drugs tested. Sequencing of the *cyp51A* gene revealed five mutations unlinked to azole resistance in all A. fumigatus isolates (17) (Table 2). Since no ECVs have been established for terbinafine or micafungin, three WT strains (ATCC 13073, Af293, and R21 [18]) were included in the study for comparison reasons. The clinical isolates showed an MIC range of 0.5 to 1 μg/ml for terbinafine compared to 0.5 to 2 μg/ml for the control strains (Table 2); hence, we considered the clinical isolates to be WT for this antifungal drug. Regarding echinocandins, isolate 24053B showed a 16.6- to 33.3- and 66.6-fold increase in minimum effective concentrations (MECs) for CSF and MCF, respectively, compared to the control strains. The other 11 isolates recovered from the patient showed a WT phenotype to all echinocandins assayed (Table 2). The reduced *in vitro* susceptibilities to echinocandin drugs were confirmed in GS enzyme assays. Product-entrapped β-(1,3)-glucan synthase complexes were extracted from the prototype WT strain ATCC 13073 and the 24053B clinical isolate, as previously described (10, 11), and the echinocandin inhibition parameter 50% inhibitory concentration (IC_50_) was determined. The *fks1*-encoded enzyme extracted from the 24053B clinical strain showed a multilog increase in IC_50_s compared to the WT (>10^6^-fold change for both CSF and MCF) (Fig. 2), indicating that it was nearly insensitive to drug. DNA sequence analysis of the published A. fumigatus Af293 *fks1* gene revealed a point mutation at nucleotide position 2072 (T to C) in the 24053B isolate. This nucleotide change conferred a Phe-to-Ser amino acid substitution in codon position 675, the first codon of the highly conserved hot spot 1 region of *fks1* (nucleotides [nt] 2071 to 3003→amino acids [aa] 675 to 684) (Table 3). The equivalent mutation in this hot spot 1 region is prominent and well known to confer echinocandin resistance in Candida albicans (F641S) and other Candida spp., and it has been linked with echinocandin clinical failure (9–11). It is noteworthy that the 24053B isolate was not recovered from any other samples collected from the patient, even though micafungin therapy failed. As has been described previously, multiple strains are present in aspergillomas, and the strain that is grown from sputum often does not reflect the full spectrum of strains present. It is likely that the patient still harbors MCF-resistant strains. The addition of terbinafine did not prevent the emergence of resistance to MCF, although it is possible it delayed its emergence.

**TABLE 2:** MIC/MEC distributions of the antifungal drugs tested in the study for the Aspergillus species clinical isolates

No. or WT^*a*^	Lab no.	Date received (day/mo/yr)	Species	MIC/MEC (mg/liter)								Cyp51A changes^*b*^	Fks1 changes^*a*^	ST
				ISA	ITR	POS	VOR	CSF	MCF	AMB	TERB			
1	22432	2/9/2010	A. fumigatus	0.25	0.25	0.12	0.25	0.12	0.03	2	1	Y46F, V172 M, T248N, E255D, K427E	ND	9
2	23525	5/4/2010	A. fumigatus	0.25	0.25	0.12	0.12	0.12	0.03	2	0.5	Y46F, V172 M, T248N, E255D, K427E	ND	9
3	24053A	6/15/2010	A. fumigatus	0.25	0.25	0.25	0.25	0.12	0.03	2	1	Y46F, V172 M, T248N, E255D, K427E	S53G	12
4	24053B	6/15/2010	A. fumigatus	0.5	0.25	0.25	1	2	2	2	1	Y46F, V172 M, T248N, E255D, K427E	S53G and F675S	9
5	24555	7/28/2010	A. fumigatus	0.25	0.25	0.25	0.25	0.12	0.03	2	0.5	Y46F, V172 M, T248N, E255D, K427E	ND	9
6	29576A	7/27/2011	A. flavus	0.25	0.5	0.25	0.25	0.12	0.03	2	0.06	WT^*c*^	ND	ND
7	29576B	7/27/2011	A. fumigatus	0.25	0.25	0.25	0.25	0.12	0.03	2	0.5	Y46F, V172 M, T248N, E255D, K427E	ND	9
8	30906	10/25/2011	A. fumigatus	0.25	0.5	0.25	0.12	0.12	0.03	2	1	Y46F, V172 M, T248N, E255D, K427E	ND	12
9	33460	4/10/2012	A. fumigatus	0.25	0.5	0.25	0.12	0.12	0.03	2	1	Y46F, V172 M, T248N, E255D, K427E	ND	9
10	45755	12/30/2013	A. fumigatus	2	1	1	0.5	0.12	0.03	2	1	Y46F, V172 M, T248N, E255D, K427E	ND	12
11	53619	1/28/2015	A. fumigatus	0.25	0.5	0.25	0.25	0.12	0.03	2	0.5	Y46F, V172 M, T248N, E255D, K427E	ND	9
12	62194	12/24/2015	A. fumigatus	0.25	0.5	0.25	0.12	0.12	0.03	2	0.5	Y46F, V172 M, T248N, E255D, K427E	ND	9
ATCC 13073			A. fumigatus	0.25	0.25	0.25	0.25	0.12	0.03	2	1	ND	S53G	ND
R21			A. fumigatus	0.25	0.25	0.25	0.25	0.12	0.03	2	2	ND	S53G	ND
Af293			A. fumigatus	0.12	0.12	0.12	0.12	0.06	0.03	1	0.5	ND	WT	ND

a The three WT strains (ATCC 13073, R21, and Af293) were included for comparison purposes.

b Reference strain used was Af293. ND, not determined.

c Reference strain used was A. flavus NRRL3357.

**TABLE 3:** *fks1* hot spot 1 sequencing of the A. fumigatus 24053B isolate

Strain	*fks1* HS1^*a*^	
	nt	aa
Af293 (reference)	T**T**CCTGACCCTGTCTTTCAAGGATCCGATCCG	**F**LTLSFKDPI
ATCC 13073	T**T**CCTGACCCTGTCTTTCAAGGATCCGATCCG	**F**LTLSFKDPI
24053B	T**C**CCTGACCCTGTCTTTCAAGGATCCGATCCG	**S**LTLSFKDPI

a HS1, hot spot 1. Nucleotide and amino acid changes have been highlighted in bold letters.

In conclusion, a resistance-associated point mutation in the well-conserved hot spot 1 region of *fks1* conferring an F675S amino acid substitution was found in A. fumigatus isolate 24053B recovered from a patient on micafungin therapy for CPA. The mutant strain yielded a β-(1,3)-glucan synthase enzyme with highly reduced (>5 to 6 log orders) sensitivity to echinocandin drugs, resulting in elevated MECs and echinocandin clinical failure. To date, this is the first reported case of echinocandin resistance due to a characteristic point mutation in the *fks1* gene in an A. fumigatus clinical isolate.
